# Who Matters For The Subjective Perceptions Toward Gerontechnology?

**DOI:** 10.1093/geroni/igab046.2497

**Published:** 2021-12-17

**Authors:** Susanna Joo, Seong Hee Kim, Changmin Lee, Chang Oh Kim, Yun Mook Lim, Hey Jung Jun

**Affiliations:** 1 Yonsei University, Seodaemun-gu, Seoul-t'ukpyolsi, Republic of Korea; 2 Sungkyunkwan University, Seoul, Republic of Korea; 3 Yonsei University, Seoul, Seoul-t'ukpyolsi, Republic of Korea; 4 Severance Hospital, Yonsei University College of Medicine, Seoul, Seoul-t'ukpyolsi, Republic of Korea

## Abstract

The present study aims to investigate how personal relationship satisfaction moderate the associations between types of social support providers and the subjective perceptions toward gerontechnology among Korean older adults. Data were collected by an online survey in February 2021. The sample was 256 older Koreans who have a partner and children (N=109 older adults with low personal relationship satisfaction, N=147 older adults with high personal relationship satisfaction, Age: 66-88, M=69.91, SD=4.19). The dependent variables for the subjective perceptions toward gerontechnology were attitude toward using gerontechnology and anxiety for gerontechnology. Independent variables were four types of social support providers (spouse, children, siblings/relatives, and friends/neighbor). Personal relationship satisfaction was a binary moderator, dividing the sample into low and high personal relationship satisfaction groups. We applied multigroup structural equation modeling. The results showed associations between social support providers and subjective perceptions toward gerontechnology differed by the quality of personal relationships. In detail, receiving support from spouses was associated with the lower level of anxiety of using gerontechnology among older adults in the low personal relationship satisfaction group. Moreover, receiving support from spouses was associated with a higher level of attitude toward using gerontechnology in the high personal relationship group. Receiving social support from other providers were not significant in both groups. The findings imply that the partner living with was salience for positive perception toward gerontechnology. Furthermore, support from spouses may differently work on the subjective perception toward gerontechnology by the quality of personal relationships.

